# The behavior of ozone on different iron oxides surface sites in water

**DOI:** 10.1038/s41598-019-50910-w

**Published:** 2019-10-14

**Authors:** Liqiang Yan, Jishuai Bing, Hecheng Wu

**Affiliations:** 1 Nanjing University of Aeronautics and Astronautics, School of Economics and Management, Nanjing, 210016 China; 2 Marine Resources Development Institute of Jiangsu, Huaihai Institute of Technology, Lianyungang, 222005 China

**Keywords:** Environmental social sciences, Environmental sciences, Environmental sciences, Environmental social sciences

## Abstract

A transformation process of ozone on different iron oxides suspensions, including α-Fe_2_O_3_, α-FeOOH, Fe_3_O_4_, was carried out using FTIR of adsorbed pyridine, ATR-FTIR and electron paramagnetic resonance (EPR) spectra with isotope ^18^O_3_. It was verified that on the surface isolated hydroxyl groups and the surface hydroxyl groups without acid sites of these iron oxides, ozone was electrostatically adsorbed and did not interact with the surface of these oxides, stably existed as ozone molecule. In contrast, ozone could replace the surface hydroxyl groups on Lewis acid sites of oxides, and directly interacted with the surface metal ions, decomposing into reactive oxygen species (ROS) and initiating the surface metal redox. The results indicate that Lewis acid sites were active center while the electronic cycle of the Fe^2+^/Fe^3+^ is advantageous to promote ozone decomposition into O_2_^•−^ and ^•^OH radicals. The mechanism of catalytic ozonation in different surface acid sites of iron oxides aqueous suspension was proposed on the basis of all experimental information.

## Introduction

Ozone is widely used in the treatment of drinking water and wastewater and the disinfection of artificial pools, etc.^1^. In practical applications, however, the utilization rate of ozone is low and pollutants cannot be completely oxidized, so ozone-based advanced oxidation technology has drawn much attention from researchers^2^. Heterogeneous catalytic ozonation is an effective technology to degrade refractory organic matter and remove residual ozone in ozone treatment water^3–5^. A variety of efficient catalysts have been developed for catalytic ozonation. However, the transformation of ozone on the micro interface of water-catalyst are still largely unknown, which is essential to optimize catalyst development and apply this technique to water treatment^6^.

It has been defined that ozone could be adsorbed on various material surfaces and was decomposed into reactive oxygen species at gas-solid phase^7^. However, John M. Roscoe observed that water molecular has more competitive ability than ozone for the surface of oxides^8^. Therefore, the transformation mechanism of ozone on catalyst suspension becomes more complex due to the presence of a large number of H_2_O molecules^6^. Metal oxides in water are first hydroxylated due to the dissociation and chemical adsorption of water molecules. Surface hydroxyl groups have ligand exchange properties with water, organic pollutants or inorganic ions, and are the main adsorption centers^9^. There are mainly three hydroxyl groups on the surface of oxides: (i) isolated hydroxyl groups; (ii) hydrogen-bonded hydroxyl groups; (iii) bridged hydroxyl groups^10–12^. Different hydroxyls would be formed on different oxides depending on the structure characterization of the oxides, resulting in different catalytic ozonation processes at the water-catalyst interface^13^. Therefore, it is essential to distinguish the different surface reaction process for defining the crucial sites of catalyst surface in catalytic ozonation process.

According to the general viewpoint in the literature of catalytic ozonation, surface hydroxyl and/or Lewis acid sites are considered ozone decomposition sites^14,15^. While other studies have found that not all hydroxyl groups have catalytic activity^16^. Moreover, surface hydroxyl groups were not clarified for the catalytic ozonation. Recently, Our results show that ozone can be adsorbed on the surface of Lewis acid competing with water and decompose to produce reactive oxygen species (ROS)^17^. Nevertheless, to date, it is still not very clear for the transformation of ozone on non-dissociated hydroxyl groups or different acid sites of metal oxides at water-solid phase.

The purpose of this study was to investigate the behavior of ozone on the surface of iron oxides and identify the key surface factors of catalysts that play a key role in ozone conversion. Three kinds of oxides, α-Fe_2_O_3_, α-FeOOH and Fe_3_O_4_, commonly used in catalytic ozonation were selected. Different iron oxides have different crystal forms and thus have different surface sites. Both the hydroxylation of these iron oxides and the adsorption of ozone were followed by *in situ* attenuated total reflection infrared (ATR-FTIR) spectroscopy, the competition of ozone and water for different surface sites was verified. Furthermore, the decomposition of ozone was determined by electron paramagnetic resonance (EPR) spectra with oxygen isotope ^18^O_3_. The activity and properties of these oxides was evaluated by ibuprofen (IBU), which is an anti-inflammatory drug, has been detected in surface water and wastewater at a range from ng to low μg L^−1^ levels due to its stability for photolysis and biodegradation. The highlight of this paper is to reveal the relationship between the mechanism of catalytic ozonation and the acidity characterization of the catalyst surface, which provides theoretical support for the development of new catalysts.

## Results and Discussion

### Characterization of catalysts

**Figure 1:**
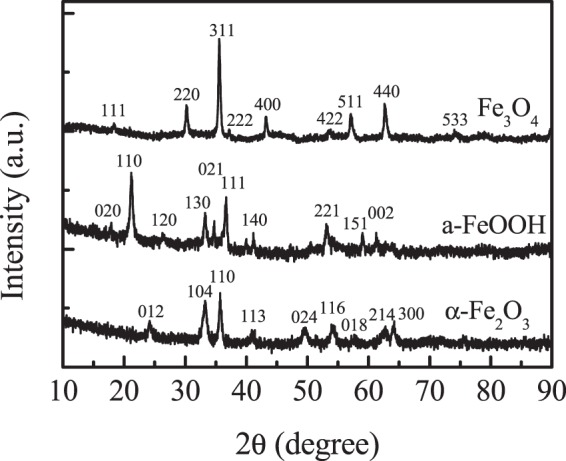
XRD patterns of α-Fe_2_O_3_, α-FeOOH and Fe_3_O_4_.

**Figure 2:**
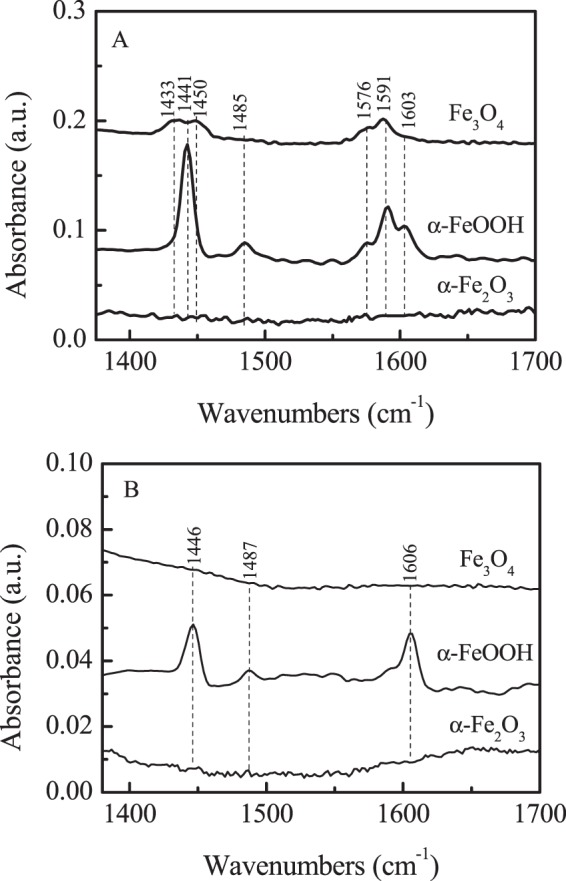
Py-FTIR for α-Fe_2_O_3_, α-FeOOH and Fe_3_O_4_ after outgassing at (**A**) 20 °C and (**B**) 150 °C.

The XRD patterns of iron oxides were shown in Fig. 1. All the samples exhibited the typical crystalline structure of α-Fe_2_O_3_ (JSPDS card 01-073-2234), α-FeOOH (JSPDS card 01-081-0463) and Fe_3_O_4_ (JSPDS card 01-088-0315), respectively, and no additional phase was contained in the samples, indicating the iron oxides were successful prepared. The nature and strength of acid sites of iron oxides were further determined by Py-FTIR after degassing at 20 °C and 150 °C (Fig. 2). In the Py-FTIR spectra, after degassing at 20 °C and 150 °C, no peak was observed for α-Fe_2_O_3_, indicating that there is no acid sites in α-Fe_2_O_3_. For Fe_3_O_4_ and α-FeOOH, six infrared absorption peaks around 1433, 1441, 1450, 1576, 1591 and 1603 cm^−1^ of Lewis acid sites appeared in the Py-FTIR spectra after degassing at 20 °C. However, all the IR absorption peaks of Fe_3_O_4_ were entirely removed at 150 °C, indicating these infrared absorption peaks are the adsorption of pyridine on weak Lewis acid sites^18,19^. While in α-FeOOH sample, the intensity of all infrared absorption peaks becomes weaker and all peaks position shifted upwards at further to evacuation up to 150 °C. This shift indicated that the species, a Lewis coordinated one, were most sensitive to coverage effects^19^. An attempt has also been made to quantitatively estimate the number of Lewis acid sites for the oxides using pyridine adsorption followed by degassing at 20 and 150 °C according to the described method (Table 1)^20^. After degassing at 20 °C, the total amount of Lewis acid sites of the Fe_3_O_4_, and α-FeOOH were 307.6 and 795.4 μmol g^−1^, respectively. After degassing at 150 °C, however, there were hardly any Lewis acid sites on the surface of Fe_3_O_4_, and the Lewis acid amount were decreased to 106.7 μmol g^−1^ for α-FeOOH, indicating that main weak Lewis acid sites were on the surface of Fe_3_O_4_, and α-FeOOH.

**Table 1:** Surface area, surface hydroxyl site density (Ns), pH_pzc_ and Lewis acid amount degassing at 20 and 150 °C of α-Fe_2_O_3_, α-FeOOH and Fe_3_O_4_.

Sample	Ns (mmol g^−1^)	pH_pzc_	Lewis acid amount (umol g^−1^)	
			20 °C	150 °C
α-Fe_2_O_3_	57	7.2	0	0
α-FeOOH	330	8.6	795.4	106.7
Fe_3_O_4_	116	6.8	307.6	0

**Figure 3:**
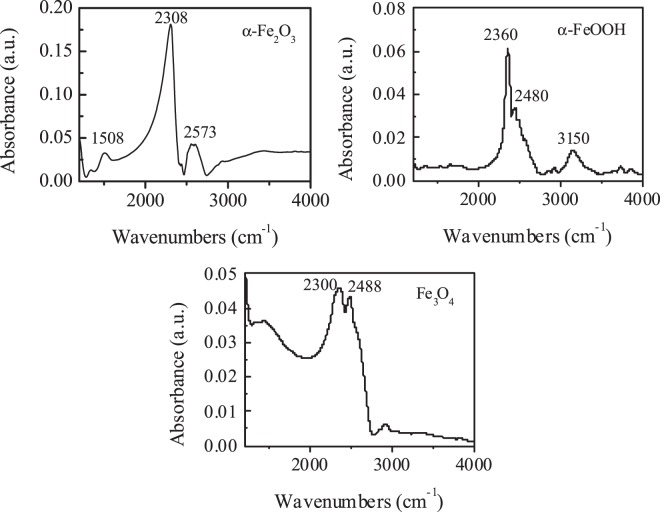
ATR-FTIR spectra of different iron oxide suspensions in D_2_O. (Catalyst concentration: 100 g L^−1^, pD: 7.0).

By a saturated deprotonation method the surface hydroxyl density (Ns) of the prepared oxides was measured. The Ns of α-Fe_2_O_3_, α-FeOOH and Fe_3_O_4_ were 57, 330, and 116 mmol g^−1^, respectively (Table 1). Furthermore, to distinguish the surface hydroxyl groups of catalyst from water, the heavy water (D_2_O) instead of H_2_O was used in ATR-FTIR experiments. As shown in Fig. 3, two hydroxyl absorbance peaks around 2308 and 2573 cm^−1^ for α-Fe_2_O_3_ were attributed to surface isolated hydroxyl and hydrogen-bonded hydroxyl groups^10,18^. The IR bands at 2300 and 2488 cm^−1^ for Fe_3_O_4_ were isolated hydroxyl and hydrogen-bonded hydroxyl on weak Lewis acid sites^21^. The IR bands at 2360 and 2480 cm^−1^ for α-FeOOH were bridged hydroxyl groups on strong Lewis acid sites and hydrogen-bonded hydroxyl on weak Lewis acid sites, respectively^22^. The surface properties of iron oxides determine the behavior of ozone at different surface sites.

### Adsorption and decomposition of ozone onto different iron oxides in water

**Figure 4:**
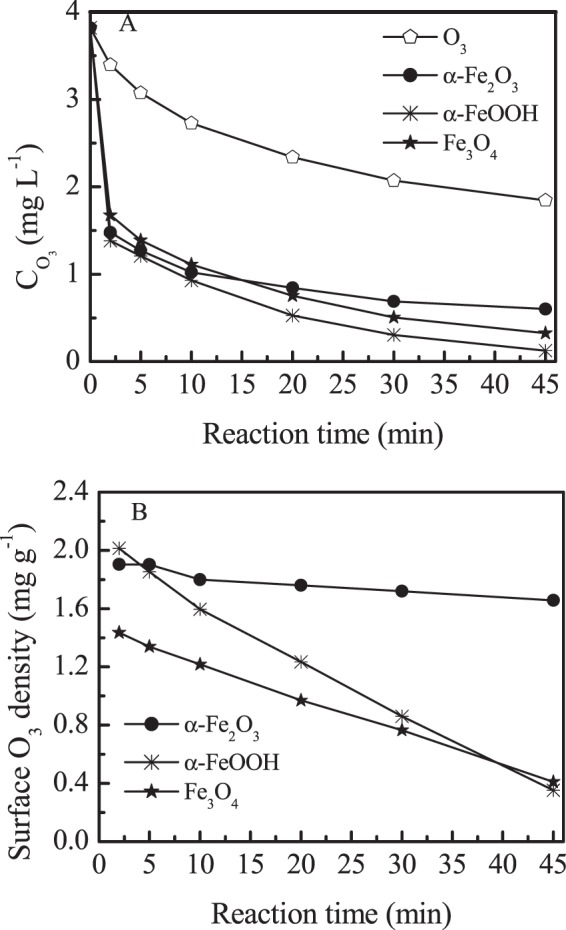
The changes of ozone concentration with time in bulk water (**A**) and the surface of iron oxides (**B**) in different processes. (Catalyst dose: 1.5 g L^−1^, initial pH: 7.0).

Surface adsorption curve of ozone on different oxides was shown in Fig. 4. The concentration of ozone decreased rapidly in oxide suspensions than O_3_ alone aqueous solution due to adsorption of the catalyst at 2 min (Fig. 4A), then the concentration of ozone continuously decreased with reaction time.α-FeOOH had the highest decay rate of ozone. Figure 4B showed the changes of ozone concentration on the surface of the oxides. At 2 min reaction time, the O_3_ adsorption amount was 1.91, 2.01 and 1.43 mg g^−1^ for α-Fe_2_O_3_, α-FeOOH and Fe_3_O_4_, respectively. Then the concentration of ozone tends to steady in α-Fe_2_O_3_ suspension after 10 min. On the contrast, the concentration of ozone continuously decreased in α-FeOOH and Fe_3_O_4_ suspensions with reaction time, indicating these two oxides contribute to the decomposition of ozone.

**Figure 5:**
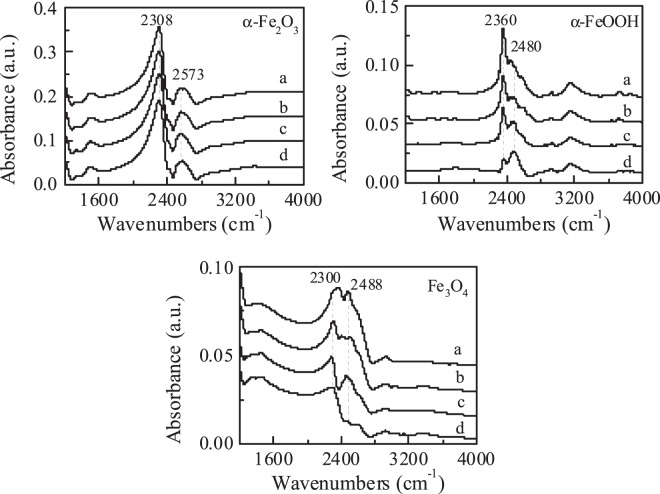
ATR-FTIR spectra of iron oxides suspended in D_2_O bubbling ozone for different time: (**a**) 0 min, (**b**) 2 min, (**c**) 5 min and (**d**) 10 min. (catalyst dose: 100 g L^−1^, pD: 7.0).

Figure 5 shows the D_2_O hydroxylation of iron oxides with bubbling ozone by *in-situ* ATR-FTIR. Obviously, in α-Fe_2_O_3_ suspension, with increasing bubbling ozone time, the intensities of the peaks of surface isolated hydroxyl (2308 cm^−1^) and hydrogen-bonded hydroxyl (2573 cm^−1^) hardly had any change, revealing that the hydroxyl groups in the non acidic sites cannot be replaced by ozone, indicating that ozone was electrostatically adsorbed on these surface hydroxyl groups. Also, in Fe_3_O_4_ suspension, the intensities of these isolated hydroxyl peaks at 2300 cm^−1^ hardly had any changed with the constant contact with ozone. The results verified that these isolated hydroxyls also could not exchange with ozone on Fe_3_O_4_. While the peak of hydrogen-bonded hydroxyl on weak Lewis acid sites at 2488 cm^−1^ for Fe_3_O_4_, bridged hydroxyl groups and hydrogen-bonded hydroxyl on Lewis acid sites at 2360 and 2480 cm^−1^ for α-FeOOH decreased gradually, indicating that the hydroxyl groups were replaced by ozone by competition with water, although water seems to be a stronger Lewis base than ozone molecule. These results show that all the isolated hydroxyls and hydrogen-bonded hydroxyl on non acidic sites cannot be substituted by ozone, while ozone can replace hydroxyl groups in Lewis acidic sites and decompose effectively. Furthermore, the pH_pzc_ of α-Fe_2_O_3_, α-FeOOH and Fe_3_O_4_ was 7.2, 8.6 and 6.8, respectively (Table 1). However, the adsorption and decomposition of ozone on the oxide surfaces was not directly related to the pH_pzc_, which only depends on the hydroxyl groups of Lewis acid sites on the surface of the oxides.

### Ozone transformation on the surface of different iron oxides

**Figure 6:**
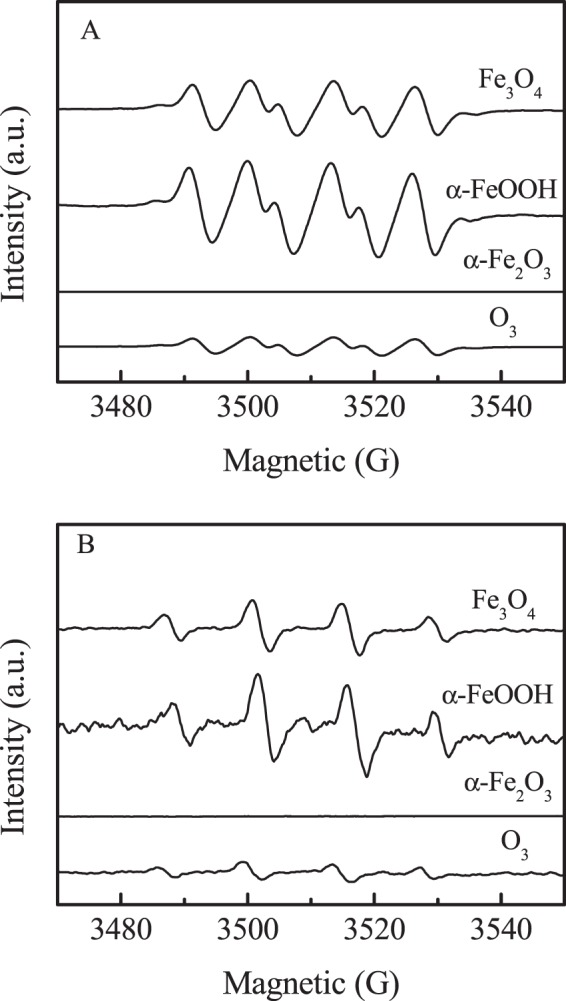
EPR signal in methanol dispersion for BMPO-HO_2_^•^/O_2_^•−^ (**A**) and aqueous dispersion for BMPO-^•^OH (**B**) with ozone.

**Figure 7:**
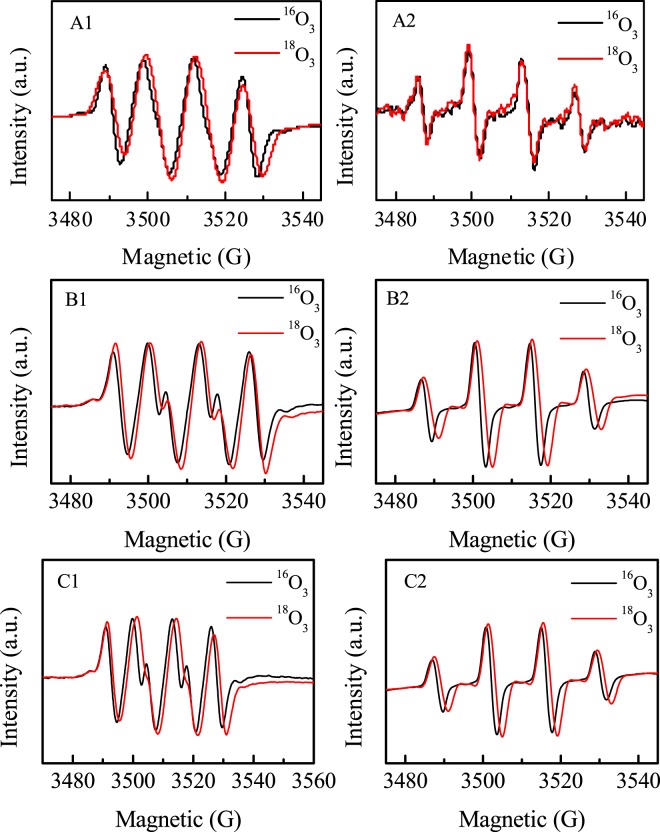
EPR spectra of O_3_ alone (**A**), α-FeOOH (**B**) and Fe_3_O_4_ (**C**) recorded in methanol dispersion for BMPO-HO_2_^•^/O_2_^•−^ (A1, B1 and C1) and aqueous dispersion for BMPO-^•^OH (A2, B2 and C2) with ^16^O_3_ or ^18^O_3_.

EPR spin-trap technique was used to analyze the ROS with ozone decomposition in these iron oxide suspensions. As illustrated in Fig. 6, neither O_2_^•−^ nor ^•^OH signals were detected in α-Fe_2_O_3_ suspension. The results confirmed that the physics-sorbed ozone was stable on the surface of α-Fe_2_O_3_, which is consistent with the results of the adsorption and decomposition of ozone on α-Fe_2_O_3_ surface in water, indicating that ozone was direct oxidant in α-Fe_2_O_3_ suspesnsion. Both higher O_2_^•−^ and ^•^OH signals were observed in α-FeOOH and Fe_3_O_4_ suspensions, and the signal strength of α-FeOOH is larger than that of Fe_3_O_4_. The results were consistent with the ozone decomposition rate in different oxides suspensions (Fig. 4), demonstrating that ozone decay was accompanied by the generation of O_2_^•−^ and ^•^OH. In order to further clarify the mechanism of O_2_^•−^ and ^•^OH generation on the surface of oxides with the decomposition of ozone, isotope ^18^O_3_ instead of ^16^O_3_ was used in EPR studies of iron oxides suspensions. Compared the EPR spectra of the ^18^O_3_ with those of ^16^O_3_, the position of BMPO-^•^OH signals did not change, while BMPO-O_2_^•−^ signal broadening in O_3_ alone aqueous solution (Fig. 7)^23^. The results confirm that reactions (1–2) are the main reactions in ozonation process^24^.

1 $$\,{}^{18}{\rm{O}}_{3}+{{\rm{HO}}}^{-}\to \,{}^{18}{\rm{O}}_{2}^{\cdot -}+{\rm{HO}}{}^{18}{\rm{O}}_{\cdot },\,\,{{\rm{k}}}_{2}=70\,{{\rm{M}}}^{-1}{{\rm{s}}}^{-1}$$

2 $$\,{}^{18}{\rm{O}}_{3}+{{\rm{HO}}}_{2}^{\cdot }\iff \,{}^{18}{\rm{O}}_{2}+\,{}^{18}{\rm{O}}{\rm{O}}+{{\rm{HO}}}^{\cdot },\,{{\rm{k}}}_{2}=1.6\times {10}^{9}{{\rm{M}}}^{-1}{{\rm{s}}}^{-1}$$

**Figure 8:**
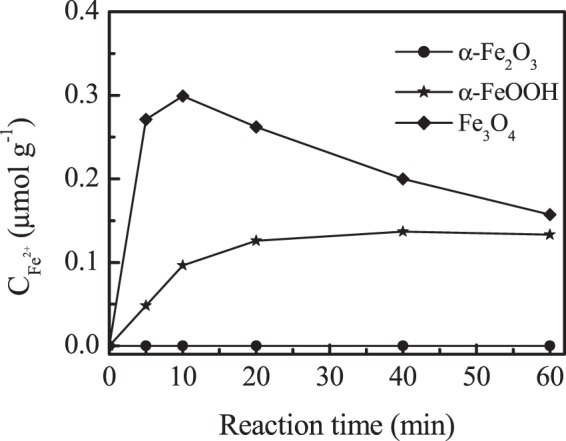
The concentration of surface Fe^2+^ on α-Fe_2_O_3_, Fe_3_O_4_ and α-FeOOH aqueous dispersions during catalytic ozonation process. (Initial pH: 7.0, catalyst concentration: 1.5 g L^−1^).

In α-FeOOH and Fe_3_O_4_ aqueous dispersion containing ^18^O_3_, both O_2_^•−^ and ^•^OH signals became broadening, indicating that the hydroxyl radical and superoxide radical originated from the decomposition of ^18^O_3_ molecule on the surface of the oxides. This may be due to the electron cycle of Fe^2+^/Fe^3+^ involved in the reaction of ozone decomposition. To ascertain the conjecture, the Fe^2+^ concentrations on the surface of α-Fe_2_O_3_, α-FeOOH and Fe_3_O_4_ at different reaction time were measured (Fig. 8). No Fe^2+^ was detected on α-Fe_2_O_3_ surface with ozone solution, confirming that Fe^2+^ was not involved in the reaction. The results indicated that the surface isolated hydroxyl groups and hydrogen-bonded hydroxyl on the surface of α-Fe_2_O_3_, blocked that ozone directly interacted with the surface Fe^3+^, keeping the adsorbed ozone stable, which was adsorbed by electrostatic forces. Differently, in α-FeOOH suspension with ozone, the produced surface Fe^2+^ concentration increased with reaction time and tended to be stable at about 20 min. In Fe_3_O_4_ suspension with ozone, there was high Fe^2+^ concentration at the beginning of the reaction, then decreased to be oxidized to Fe^3+^ with prolonged reaction time. The results indicated that the adsorbed ozone on the Lewis acid sites directly reacted with the surface Fe^3+^ of α-FeOOH and Fe_3_O_4_, enhancing the formation of ^•^OH and O_2_^•−^ radicals. In light of the experimental data, a mechanism scheme is proposed for the ozone decomposition at the Lewis acids of α-FeOOH and Fe_3_O_4_. The main reactions are shown in Eqs (3–5).

3 $$\equiv {{\rm{Fe}}}^{3+}-{\rm{OH}}+{}^{18}{\rm{O}}_{3}\to \equiv {{\rm{Fe}}}^{3+}-{}^{18}{\rm{O}}_{3}+{{\rm{OH}}}^{-}$$

4 $$\equiv {{\rm{Fe}}}^{3+}-{}^{18}{\rm{O}}_{3}+{{\rm{OH}}}^{-}\to \equiv {{\rm{Fe}}}^{2+}+{\rm{HO}}{}^{18}{\rm{O}}_{\cdot }+{}^{18}{\rm{O}}_{2}$$

5 $$\equiv {{\rm{Fe}}}^{2+}+{}^{18}{\rm{O}}_{3}+{{\rm{H}}}_{2}{\rm{O}}\to \equiv {{\rm{Fe}}}^{3+}-{\rm{OH}}+{}^{\cdot 18}{\rm{O}}{\rm{H}}+{}^{18}{\rm{O}}_{2}$$

### Catalytic ozonation of ibuprofen in different iron oxide suspensions

**Figure 9:**
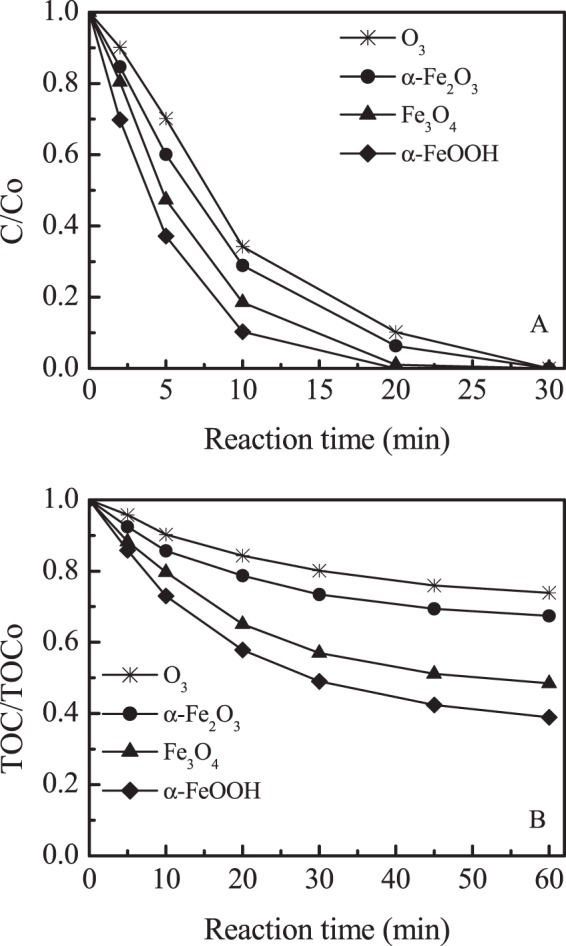
(**A**) Catalytic ozonation of IBU in various suspensions. (**B**) TOC removal. (Initial pH = 7.0, initial IBU concentration = 10 mg L^−1^, catalyst concentration = 1.5 g L^−1^, gaseous ozone concentration = 30 mg L^−1^).

The catalytic activity of the oxides was evaluated by ibuprofen. The adsorption removal of ibuprofen by α-Fe_2_O_3_, Fe_3_O_4_ and α-FeOOH were about 5%, 3% and 6% in equilibrium, respectively (Fig. S1). The comparison of ibuprofen and TOC removal among different iron oxide suspensions were shown in Fig. 9. The presence of catalyst was advantageous for ibuprofen degradation compared with ozonation alone, and α-FeOOH had the highest catalytic activity. The ozonation of ibuprofen led to 26% TOC removal at 60 min. The simultaneous use of ozone and α-Fe_2_O_3_ slightly increased ibuprofen removal, TOC removal was increased to 32%. In the α-FeOOH /O_3_ process, a maximum of 62% TOC removal was obtained at 60 min oxidation time, while about 52% of TOC were removed at the same time in Fe_3_O_4_/O_3_ process. Because only 6% TOC removal by α-FeOOH adsorption. It indicated that there was a significant synergetic effect (between α-FeOOH adsorption and ozonation alone in α-FeOOH /O_3_ process). Furthermore, the catalytic activity of different iron oxides was consistent with the amount of surface Lewis acid site. The results indicate that Lewis acid sites were active center, which causes the effective decomposition of ozone. While the electronic cycle of the Fe^2+^/Fe^3+^ was advantageous to promote ozone decomposition into O_2_^•−^ and ^•^OH radicals, leading to effective degradation and mineralization of ibuprofen. These finding provided a new sight for the mechanism of catalytic ozonation and the design of new type heterogeneous catalytic ozonation catalyst.

## Conclusions

Three iron oxides were prepared, which are α-Fe_2_O_3_, α-FeOOH and Fe_3_O_4_. These iron oxides have different acid sites and thus different hydroxyl groups. α-Fe_2_O_3_ has no acid sites. Ozone was electrostatic adsorbed stably at isolated hydroxyl and hydrogen-bonded hydroxyl on α-Fe_2_O_3_, and did not react with the surface Fe^3+^ ions due to blocking by these hydroxyl groups. Different mechanisms were observed for α-FeOOH and Fe_3_O_4_, ozone was adsorbed on the surface Lewis acid sites of α-FeOOH and Fe_3_O_4_ competing with water, directly interacted with the surface Fe^3+^ ions and mainly converted into O_2_^•−^ and ^•^OH due to Fe^2+^/Fe^3+^ electronic circulation when ozone adsorbed on α-FeOOH and Fe_3_O_4_.

## Experimental Section

### Preparation of catalysts

The α-FeOOH particles were prepared by adding 1.0 mol L^−1^ sodium hydroxide solution to 0.1 mol L^−1^ferric nitrate solution containing 20 mol % amounts of sodium citrate up to pH 12, in a polypropylene screwcapped vessel at room temperature. The resulting precipitates were aged for 4 days in a thermostat at 30 °C. The precipitates were thoroughly washed with water and dried in air at 70 °C for 16 h^25^. Magnetite Fe_3_O_4_ was prepared from the precipitate of ferric nitrate solution with aqueous sodium hydroxide^26^. The precipitate was washed with ammonium acetate solution, dried at 100 °C for 12 h, and treated at 400 °C under an atmosphere of N_2_ for 2 h. α-Fe_2_O_3_ was synthesized by calcining a precursor of ferrous oxalate, which was precipitated from a Fe^2+^ solution mixed with oxalic acid^27^.

### Characterization

Powder X-ray diffraction (XRD) of the catalyst was analyzed on a Scintag-XDS-2000 diffractometer with Cu Kα radiation (λ = 1.540598 Å). BET-surface areas were measured by N_2_ adsorption using a Micromeritics ASAP2020 automated gas sorption system (USA). Infrared spectra of adsorbed pyridine (Py-FTIR) were taken on a Bruker Tensor 27 FT-IR spectrometer. The samples were pressed into self-supporting wafers and were evacuated in a vacuum cell at 100 °C for 2 h. The infrared spectra of adsorbed pyridine were recorded after degassing at 20 °C and 150 °C. The point of zero charge (pH_pzc_) of the catalysts was determined with a Zetasizer Nano (Malvern, UK). The surface hydroxyl density (Ns) of catalysts was measured according to a saturated deprotonation method^28^.

### Experimental procedures

The concentration of ozone in the process of catalytic ozonation and catalyst surface was analyzed by improved indigo method^29^. Ozone decomposition experiments were carried out in a 250 mL three-mouth flask at 20 °C. Ozone was generated by a 3S-A5 laboratory ozone generator (Tonglin Technology, China). Firstly, ozone was continuously bubbled into 250 mL of water to get ozone saturated aqueous solution (3.82 mg L^−1^). After 0.375 g of catalyst was added, the time counting was immediately started. Addition of catalysts almost had no effect on the solution pH (7 ± 0.1). At given time intervals, 1 mL suspensions were collected and 1 mL filtrate was obtained at the same time, then it was added into 8 mL indigo solution respectively. The samples were mixed, filtered through a 0.22 µm Millipore filter for ozone concentration measurement. In the determination experiment, all catalysts hardly adsorb any indigo, and in order to reduce the impact of filtration on aqueous ozone, 60 mL of the ozone stock solution was pressed through the filter before sample filtration, so the ozone concentration was not affected by the filtration process.

The catalytic reaction procedure was carried out in a 1.2 L column reactor at 20 °C. In a typical procedure, 1 L ibuprofen around 10 mg L^−1^ aqueous solution and 1.5 g of catalyst powder were mixed in the reactor under continuously magnetically stir. Ozone was produced *in situ* from pure oxygen by a 3S-A5 laboratory ozone generator (Tonglin Technology, China). Gaseous O_3_ (30 mg L^−1^) was continuously bubbled into the reactor through the porous plate of the reactor bottom at a 200 mL min^−1^ flow rate. The excess ozone in the outlet gas was trapped by a Na_2_S_2_O_3_ solution. The same procedures were carried out for the control experiments of ozone alone and sorption without ozone. Water samples were taken at regular intervals. A 0.1 mol L^−1^ Na_2_S_2_O_3_ solution was used to quench the continuous ozonation reaction in the new withdrawn water samples and then water samples were filtered by a 0.45 μm Millipore filter to analyze ibuprofen and total organic carbon (TOC) concentrations.

### Analytical methods

The ozone concentration in the gas phase is determined by an IDEAL-2000 ozone concentration detector (China). The concentration of ibuprofen was analyzed by means of an Agilent 1200 series HPLC equipped with a UV detector at 220 nm and a ZORBAX Eclipse XDB-C18 column (4.6 × 150 mm, 5 μm). Total organic carbon (TOC) was measured by a Shimadzu TOC-V_CPH_ analyzer. Electron paramagnetic resonance (EPR) spectra were recorded at 20 °C on a Bruker A300-10/12 EPR spectrometer using 5-tert-Butoxycarbonyl-5-methyl-1-pyrroline-N-oxide (BMPO) as a spin trap agent. The ATR-FTIR spectra were measured with a Nicolet 8700 FTIR spectrophotometer (Thermo Fisher Scientific Inc., USA) equipped with a Universal ATR accessory. The ATR-FTIR samples were prepared as described previously^22^. The Fe^2+^ concentrations on the surface of α-Fe_2_O_3_, α-FeOOH and Fe_3_O_4_ at different reaction time were measured by a modified 1,10-phenanthroline method at a wavelength of 510 nm using a UV-vis spectrophotometer (U-3900, HITACHI). The 1, 10-Phenanthroline could take up Fe^2+^ from the surface of the solid phase via a specific chelating reaction^30^. In a typical process, the catalyst dispersions under different reaction time were filtered and the resulting solid was re-suspended in 10 mL of 1, 10-phenanthroline solution (1 g L^−1^) and reaction for 10 min, the new dispersion was filtered and the filtrates were analyzed at λ = 510 nm, which is the maximal adsorption wavelength for Fe^2+^-1,10-phenanthroline complex.

## Supplementary information

SUPPLEMENTARY INFO
