# Supplementary material for: The behavior of ozone on different iron oxides surface sites in water
Source: Sci Rep. 2019 Oct 14;9:14752. doi: 10.1038/s41598-019-50910-w (PMC6791862; doi:10.1038/s41598-019-50910-w)
Supplement: Supplementary file 1 — SUPPLEMENTARY INFO [file 41598_2019_50910_MOESM1_ESM.doc]

***Supporting Information***

**The behavior of ozone on different iron oxides surface sites in water**

Liqiang Yan^†^, Jishuai Bing*^,‡^, Hecheng Wu*^,†^

^†^ Nanjing University of Aeronautics and Astronautics, School of Economics and Management, Nanjing 210016, China.

^‡^ Marine Resources Development Institute of Jiangsu, Huaihai Institute of Technology, Lianyungang 222005, China.

*Corresponding author. Tel: +8617705135355; E-mail:lygylq@126.com

Pages: 2 and Figure: 1

**Fig. S1.** Comparison of IBU removal by adsorption processes in various suspensions. (Initial pH = 7.0, initial IBU concentration = 10 mg L^-1^, catalyst concentration = 1.5 g L^-1^).
